# Supplementary material for: The ΦBT1 large serine recombinase catalyzes DNA integration at pseudo-attB sites in the genus Nocardia
Source: PeerJ. 2018 May 4;6:e4784. doi: 10.7717/peerj.4784 (PMC5937489; doi:10.7717/peerj.4784)
Supplement: Supplemental Information 1 [file peerj-06-4784-s001.docx]

Table S1. Strains used in this study

| **Strain** | **Features** | **Source/Reference** |
| --- | --- | --- |
| *Nocardia brasiliensis* AUSMDU00012716 | Patient isolate | This study |
| *Nocardia terpenica* AUSMDU00012715 | Patient isolate | This study |
| *Nocardia uniformis* AUSMDU00012718 | Patient isolate | This study |
| *Nocardia arthritidis* AUSMDU00012717 | Patient isolate | This study |
| *E. coli* DH10B | F– mcrAΔ(mrr-hsdRMS-mcrBC) Φ80lacZ ΔM15 ΔlacX74 recA1 endA1 araD139 Δ(araleu)7697 galU galK rpsL nupG λ– | NEB |
| *E. coli* ET12567 (pUZ8002) | Donor strain for conjugation of DNA into *Streptomyces*. Kan^R^ Cml^R^ | (Kieser et al. 2000) |
| *Streptomyces coelicolor* A095 |  | Gift from P. Leadlay |
| *Streptomyces lividans* TK24 |  | Gift from P. Leadlay |
| *Streptomyces cinnamonensis* |  | Gift from P. Leadlay |
| *Streptomyces albus* J1074 |  | Gift from P. Leadlay |
