# Supplementary material for: The ΦBT1 large serine recombinase catalyzes DNA integration at pseudo-attB sites in the genus Nocardia
Source: PeerJ. 2018 May 4;6:e4784. doi: 10.7717/peerj.4784 (PMC5937489; doi:10.7717/peerj.4784)
Supplement: Supplemental Information 2 [file peerj-06-4784-s002.docx]

Table S2. Concordance of SRA read number with species and *attB* site, as referred to in the manuscript.

| SRA sample name | SRA accession number/link | Species/Corresponding *attB* site |
| --- | --- | --- |
| AUSMDU00012715-pRT801-2 | [SRX3735211](https://www.ncbi.nlm.nih.gov/sra/SRX3735211%5baccn%5d) | *Nocardia terpenica*, Nt-14 |
| AUSMDU00012715-pRT801-3 | [SRX3735255](https://www.ncbi.nlm.nih.gov/sra/SRX3735255%5baccn%5d) | *Nocardia terpenica*, Nt-10 |
| AUSMDU00012715-pRT801-4 | [SRX3735227](https://www.ncbi.nlm.nih.gov/sra/SRX3735227%5baccn%5d) | *Nocardia terpenica*, Nt-11 |
| AUSMDU00012715-pRT801-30 | [SRX3735226](https://www.ncbi.nlm.nih.gov/sra/SRX3735226%5baccn%5d) | *Nocardia terpenica*, Nt-17 |
| AUSMDU00012715-pRT801-A5 | [SRX3735267](https://www.ncbi.nlm.nih.gov/sra/SRX3735267%5baccn%5d) | *Nocardia terpenica*, Nt-3 |
| AUSMDU00012715-pRT801-B | [SRX3921667](https://www.ncbi.nlm.nih.gov/sra/SRX3921667%5baccn%5d) | *Nocardia terpenica*, Nt-17 |
| AUSMDU00012715-pRT801-B1 | [SRX3735261](https://www.ncbi.nlm.nih.gov/sra/SRX3735261%5baccn%5d) | *Nocardia terpenica*, Nt-1 |
| AUSMDU00012715-pRT801-C | [SRX3921666](https://www.ncbi.nlm.nih.gov/sra/SRX3921666%5baccn%5d) | *Nocardia terpenica*, Nt-5 |
| AUSMDU00012715-pRT801-D1 | [SRX3735260](https://www.ncbi.nlm.nih.gov/sra/SRX3735260%5baccn%5d) | *Nocardia terpenica*, Nt-16 |
| AUSMDU00012715-pRT801-E3 | [SRX3735242](https://www.ncbi.nlm.nih.gov/sra/SRX3735242%5baccn%5d) | *Nocardia terpenica*, Nt-3 |
| AUSMDU00012715-pRT801-H2 | [SRX3735241](https://www.ncbi.nlm.nih.gov/sra/SRX3735241%5baccn%5d) | *Nocardia terpenica*, Nt-2 |
| AUSMDU00012715-pRT801-H4 | [SRX3735240](https://www.ncbi.nlm.nih.gov/sra/SRX3735240%5baccn%5d) | *Nocardia terpenica*, Nt-17 |
| AUSMDU00012715-pRT801-I1 | [SRX3735239](https://www.ncbi.nlm.nih.gov/sra/SRX3735239%5baccn%5d) | *Nocardia terpenica*, Nt-17 |
| AUSMDU00012715-pRT801-I3 | [SRX3735238](https://www.ncbi.nlm.nih.gov/sra/SRX3735238%5baccn%5d) | *Nocardia terpenica*, Nt-4 |
| AUSMDU00012715-pRT801-K3 | [SRX3735236](https://www.ncbi.nlm.nih.gov/sra/SRX3735236%5baccn%5d) | *Nocardia terpenica*, Nt-10 |
| AUSMDU00012715-pRT801-M1 | [SRX3735235](https://www.ncbi.nlm.nih.gov/sra/SRX3735235%5baccn%5d) | *Nocardia terpenica*, Nt-13 |
| AUSMDU00012715-pRT801-N8 | [SRX3735216](https://www.ncbi.nlm.nih.gov/sra/SRX3735216%5baccn%5d) | *Nocardia terpenica*, Nt-7 |
| AUSMDU00012715-pRT801-N9 | [SRX3735217](https://www.ncbi.nlm.nih.gov/sra/SRX3735217%5baccn%5d) | *Nocardia terpenica*, Nt-15 |
| AUSMDU00012715-pRT801-N21 | [SRX3735214](https://www.ncbi.nlm.nih.gov/sra/SRX3735214%5baccn%5d) | *Nocardia terpenica*, Nt-18 |
| AUSMDU00012715-pRT801-O2 | [SRX3735215](https://www.ncbi.nlm.nih.gov/sra/SRX3735215%5baccn%5d) | *Nocardia terpenica*, Nt-8 |
| AUSMDU00012715-pRT801-O17 | [SRX3735220](https://www.ncbi.nlm.nih.gov/sra/SRX3735220%5baccn%5d) | *Nocardia terpenica*, Nt-3 |
| AUSMDU00012715-pRT801-P11 | [SRX3735221](https://www.ncbi.nlm.nih.gov/sra/SRX3735221%5baccn%5d) | *Nocardia terpenica*, Nt-3 |
| AUSMDU00012715-pRT801-P24 | [SRX3735218](https://www.ncbi.nlm.nih.gov/sra/SRX3735218%5baccn%5d) | *Nocardia terpenica*, Nt-19 |
| AUSMDU00012715-pRT801-P27 | [SRX3735219](https://www.ncbi.nlm.nih.gov/sra/SRX3735219%5baccn%5d) | *Nocardia terpenica*, Nt-6 |
| AUSMDU00012715-pRT801-P32 | [SRX3735222](https://www.ncbi.nlm.nih.gov/sra/SRX3735222%5baccn%5d) | *Nocardia terpenica*, Nt-12 |
| AUSMDU00012715-pRT801-Q6 | [SRX3735223](https://www.ncbi.nlm.nih.gov/sra/SRX3735223%5baccn%5d) | *Nocardia terpenica*, Nt-9 |
| AUSMDU00012715-pRT801-Q8 | [SRX3735244](https://www.ncbi.nlm.nih.gov/sra/SRX3735244%5baccn%5d) | *Nocardia terpenica*, Nt-6 |
| AUSMDU00012716-pRT801-A1 | [SRX3735243](https://www.ncbi.nlm.nih.gov/sra/SRX3735243%5baccn%5d) | *Nocardia brasiliensis*, Nb-1 |
| AUSMDU00012716-pRT801-A3 | [SRX3735246](https://www.ncbi.nlm.nih.gov/sra/SRX3735246%5baccn%5d) | *Nocardia brasiliensis*, Nb-2 |
| AUSMDU00012716-pRT801-A5 | [SRX3735245](https://www.ncbi.nlm.nih.gov/sra/SRX3735245%5baccn%5d) | *Nocardia brasiliensis*, Nb-3 |
| AUSMDU00012716-pRT801-A13 | [SRX3735248](https://www.ncbi.nlm.nih.gov/sra/SRX3735248%5baccn%5d) | *Nocardia brasiliensis*, Nb-1 |
| AUSMDU00012716-pRT801-B4 | [SRX3735247](https://www.ncbi.nlm.nih.gov/sra/SRX3735247%5baccn%5d) | *Nocardia brasiliensis*, Nb-4 |
| AUSMDU00012716-pRT801-B5 | [SRX3735250](https://www.ncbi.nlm.nih.gov/sra/SRX3735250%5baccn%5d) | *Nocardia brasiliensis*, Nb-5 |
| AUSMDU00012716-pRT801-C6 | [SRX3735249](https://www.ncbi.nlm.nih.gov/sra/SRX3735249%5baccn%5d) | *Nocardia brasiliensis*, Nb-6 |
| AUSMDU00012716-pRT801-C8 | [SRX3735252](https://www.ncbi.nlm.nih.gov/sra/SRX3735252%5baccn%5d) | *Nocardia brasiliensis*, Nb-4 |
| AUSMDU00012716-pRT801-C12 | [SRX3735251](https://www.ncbi.nlm.nih.gov/sra/SRX3735251%5baccn%5d) | *Nocardia brasiliensis*, Nb-7 |
| AUSMDU00012716-pRT801-D1 | [SRX3735228](https://www.ncbi.nlm.nih.gov/sra/SRX3735228%5baccn%5d) | *Nocardia brasiliensis*, Nb-4 |
| AUSMDU00012716-pRT801-E1 | [SRX3735268](https://www.ncbi.nlm.nih.gov/sra/SRX3735268%5baccn%5d) | *Nocardia brasiliensis*, Nb-8 |
| AUSMDU00012716-pRT801-F1 | [SRX3735213](https://www.ncbi.nlm.nih.gov/sra/SRX3735213%5baccn%5d) | *Nocardia brasiliensis*, Nb-9 |
| AUSMDU00012717-pRT801-A9 | [SRX3735234](https://www.ncbi.nlm.nih.gov/sra/SRX3735234%5baccn%5d) | *Nocardia arthritidis*, Na-1 |
| AUSMDU00012717-pRT801-B9 | [SRX3735233](https://www.ncbi.nlm.nih.gov/sra/SRX3735233%5baccn%5d) | *Nocardia arthritidis*, Na-2 |
| AUSMDU00012717-pRT801-C5 | [SRX3735210](https://www.ncbi.nlm.nih.gov/sra/SRX3735210%5baccn%5d) | *Nocardia arthritidis*, Na-3 |
| AUSMDU00012717-pRT801-D1 | [SRX3735253](https://www.ncbi.nlm.nih.gov/sra/SRX3735253%5baccn%5d) | *Nocardia arthritidis*, Na-2 |
| AUSMDU00012717-pRT801-D5 | [SRX3735262](https://www.ncbi.nlm.nih.gov/sra/SRX3735262%5baccn%5d) | *Nocardia arthritidis*, Na-2 |
| AUSMDU00012717-pRT801-D12 | [SRX3735259](https://www.ncbi.nlm.nih.gov/sra/SRX3735259%5baccn%5d) | *Nocardia arthritidis*, Na-4 |
| AUSMDU00012717-pRT801-E3 | [SRX3735258](https://www.ncbi.nlm.nih.gov/sra/SRX3735258%5baccn%5d) | *Nocardia arthritidis*, Na-4 |
| AUSMDU00012717-pRT801-E5 | [SRX3735257](https://www.ncbi.nlm.nih.gov/sra/SRX3735257%5baccn%5d) | *Nocardia arthritidis*, Na-5 |
| AUSMDU00012717-pRT801-E10 | [SRX3735256](https://www.ncbi.nlm.nih.gov/sra/SRX3735256%5baccn%5d) | *Nocardia arthritidis*, Na-1 |
| AUSMDU00012717-pRT801-1 | [SRX3735266](https://www.ncbi.nlm.nih.gov/sra/SRX3735266%5baccn%5d) | *Nocardia uniformis*, Nu-1 |
| AUSMDU00012717-pRT801-2 | [SRX3735265](https://www.ncbi.nlm.nih.gov/sra/SRX3735265%5baccn%5d) | *Nocardia uniformis*, Nu-1 |
| AUSMDU00012717-pRT801-3 | [SRX3735264](https://www.ncbi.nlm.nih.gov/sra/SRX3735264%5baccn%5d) | *Nocardia uniformis*, Nu-2 |
| AUSMDU00012717-pRT801-4 | [SRX3735263](https://www.ncbi.nlm.nih.gov/sra/SRX3735263%5baccn%5d) | *Nocardia uniformis*, Nu-1 |
| AUSMDU00012717-pRT801-5 | [SRX3735270](https://www.ncbi.nlm.nih.gov/sra/SRX3735270%5baccn%5d) | *Nocardia uniformis*, Nu-1 |
| AUSMDU00012717-pRT801-7 | [SRX3735269](https://www.ncbi.nlm.nih.gov/sra/SRX3735269%5baccn%5d) | *Nocardia uniformis*, Nu-1 |
| AUSMDU00012717-pRT801-9 | [SRX3735212](https://www.ncbi.nlm.nih.gov/sra/SRX3735212%5baccn%5d) | *Nocardia uniformis*, Nu-1 |
| AUSMDU00012718-pRT801-C1 | [SRX3735254](https://www.ncbi.nlm.nih.gov/sra/SRX3735254%5baccn%5d) | *Nocardia uniformis*, Nu-1 |
| AUSMDU00012718-pRT801-E1 | [SRX3735229](https://www.ncbi.nlm.nih.gov/sra/SRX3735229%5baccn%5d) | *Nocardia uniformis*, Nu-1 |
| AUSMDU00012718-pRT801-F1 | [SRX3735230](https://www.ncbi.nlm.nih.gov/sra/SRX3735230%5baccn%5d) | *Nocardia uniformis*, Nu-1 |
| AUSMDU00012718-pRT801-F2 | [SRX3735231](https://www.ncbi.nlm.nih.gov/sra/SRX3735231%5baccn%5d) | *Nocardia uniformis*, Nu-1 |
| AUSMDU00012718-pRT801-F3 | [SRX3735232](https://www.ncbi.nlm.nih.gov/sra/SRX3735232%5baccn%5d) | *Nocardia uniformis*, Nu-1 |
| AUSMDU00012718-pRT801-G1 | [SRX3735224](https://www.ncbi.nlm.nih.gov/sra/SRX3735224%5baccn%5d) | *Nocardia uniformis*, Nu-1 |
| AUSMDU00012718-pRT801-G2 | [SRX3735225](https://www.ncbi.nlm.nih.gov/sra/SRX3735225%5baccn%5d) | *Nocardia uniformis*, Nu-1 |
