# Supplementary material for: The ΦBT1 large serine recombinase catalyzes DNA integration at pseudo-attB sites in the genus Nocardia
Source: PeerJ. 2018 May 4;6:e4784. doi: 10.7717/peerj.4784 (PMC5937489; doi:10.7717/peerj.4784)
Supplement: Supplemental Information 3 [file peerj-06-4784-s003.docx]

Table S3. Insertion sites in *N. brasiliensis*, *N. arthritidis* and *N. uniformis* genomes and comparison with the *S. coelicolor* *attB* site.

| Insert name | Identity to *attB* (%)^a^ | Identity to minimal *attB* (%)^b^ | Identity with *attB*-*attP* site^c^ | Insertion events |
| --- | --- | --- | --- | --- |
| Nb-1 | 22 (30%) | 15 (41%) | 5 | 2 |
| Nb-2 | 29 (40%) | 17 (47%) | 5 | 1 |
| Nb-3 | 24 (33%) | 12 (33%) | 5 | 1 |
| Nb-4 | 28 (38%) | 17 (47%) | 5 | 3 |
| Nb-5 | 27 (37%) | 12 (33%) | 6 | 1 |
| Nb-6 | 23 (31%) | 13 (36%) | 2 | 1 |
| Nb-7 | 34 (46%) | 19 (52%) | 5 | 1 |
| Nb-8 | 34 (46%) | 17 (47%) | 5 | 1 |
| Nb-9 | 22 (30%) | 13 (36%) | 5 | 1 |
| Na-1 | 25 (34%) | 15 (41%) | 4 | 2 |
| Na-2 | 30 (41%) | 16 (44%) | 5 | 3 |
| Na-3 | 31 (42%) | 21 (58%) | 4 | 1 |
| Na-4 | 31 (42%) | 18 (50%) | 6 | 2 |
| Na-5 | 30 (41%) | 21 (58%) | 4 | 1 |
| Nu-1 | 35 (48%) | 22 (61%) | 6 | 14 |
| Nu-2 | 26 (36%) | 17 (47%) | 4 | 1 |

^a^ Number of identical nucleotide positions to the 73 bp canonical *S. coelicolor* ΦBT1 *attB* site;
^b^ Number of identical nucleotide positions to the 36 bp minimal *S. coelicolor* ΦBT1 *attB* site;
^c^ Number of identical nucleotide positions to the 9 bp *S. coelicolor attB-* ΦBT1 *attP* recombination site (Gregory et al. 2003).
