# Supplementary material for: The ΦBT1 large serine recombinase catalyzes DNA integration at pseudo-attB sites in the genus Nocardia
Source: PeerJ. 2018 May 4;6:e4784. doi: 10.7717/peerj.4784 (PMC5937489; doi:10.7717/peerj.4784)
Supplement: Supplemental Information 4 [file peerj-06-4784-s004.docx]

Table S4. *Streptomyces* genomes analysed for ΦBT1 *attB* sites.

| Strain | Identity to *attB* (%)^a^ | Identity to minimal *attB* (%)^b^ | NCBI accession, reference |
| --- | --- | --- | --- |
| *S. lividans* TK24 | 73 (100) | 36 (100) | CP009124.1, (Ruckert et al. 2015) |
| *S. ambofaciens* DSM40697 | 73 (100) | 36 (100) | CP012949.1, (Thibessard & Leblond 2016) |
| *S. ghanaensis* | 70 (95.8) | 34 (94.4) | EU368672, (Ostash et al. 2009) |
| *S. davawensis* JCM4913 | 70 (95.8) | 34 (94.4) | HE971709.1, (Jankowitsch et al. 2012) |
| *S. pactum* ACT12 | 69 (94.5) | 34 (94.4) | NZ_LIQD00000000, Unpublished |
| *S. lincolnensis* NRRL2936 | 69 (94.5) | 33 (91.7) | CP016438.1, Unpublished |
| *S. cinnamonensis* | 67 (91.8) | 33 (91.7) | -, This study |
| *S. avermitilis* NBRC14893 | 67 (91.8) | 33 (91.7) | NC_003155.5  , (Omura et al. 2001) |
| *S. griseus* NBRC13350 | 67 (91.8) | 33 (91.7) | NC_010572.1,  (Ohnishi et al. 2008) |
| *S. alboflavus* MDJK44 | 66 (90.4) | 33 (91.7) | CP021748.1, Unpublished |
| *S. violaceoruber* S21 | 65 (89) | 33 (91.7) | CP020570.1, Unpublished |
| *S. scabiei* 87.22 | 64 (87.6) | 33 (91.7) | NC_013929.1, (Bignell et al. 2010) |
| *S. niveus* SCSIO3406 | 63 (86.3) | 31 (86.1) | NZ_CP018047.1, Unpublished |
| *S. venezuelae* ATCC10712 | 63 (86.3) | 30 (83.3) | LN881739.1, (He et al. 2016) |
| *S. clavuligerus* F613-1 | 63 (86.3) | 31 (86.1) | NZ_CP016559.1, Unpublished |
| *S. bingchenggensis* BCW-1 | 62 (84.9) | 29 (80.6) | NC_016582.1, (Wang et al. 2010) |
| *S. albus* J1074 | 60 (82.2) | 28 (77.8) | NC_020990.1, (Zaburannyi et al. 2014) |
| *S. hygroscopicus* XM201 | 60 (82.2) | 29 (80.6) | CP018627.1, Unpublished |
| *S. rapamycinicus* NRRL5491 | 60 (82.2) | 29 (80.6) | CP006567.1, (Baranasic et al. 2013) |
| *S. albulus* ZPM | 59 (80.8) | 29 (80.6) | CP006871.1, (Wang et al. 2015) |
| *S. lydicus* 103 | 58 (79.5) | 27 (75) | CP017157.1, Unpublished |
| *S. fradiae* Olg4R | 54 (74) | 26 (72.2) | NZ_MCNU00000000, Unpublished |

^a^ Number of identical nucleotide positions to the 73 bp canonical *S. coelicolor* ΦBT1 *attB* site;
^b^ Number of identical nucleotide positions to the 36 bp minimal *S. coelicolor* ΦBT1 *attB* site.

**References**:

Baranasic D, Gacesa R, Starcevic A, Zucko J, Blazic M, Horvat M, Gjuracic K, Fujs S, Hranueli D, Kosec G, Cullum J, and Petkovic H. 2013. Draft Genome Sequence of Streptomyces rapamycinicus Strain NRRL 5491, the Producer of the Immunosuppressant Rapamycin. *Genome Announcements* 1. 10.1128/genomeA.00581-13

Bignell DR, Seipke RF, Huguet-Tapia JC, Chambers AH, Parry RJ, and Loria R. 2010. Streptomyces scabies 87-22 contains a coronafacic acid-like biosynthetic cluster that contributes to plant-microbe interactions. *Molecular Plant-Microbe Interactions* 23:161-175. 10.1094/MPMI-23-2-0161

He J, Sundararajan A, Devitt NP, Schilkey FD, Ramaraj T, and Melancon CE, 3rd. 2016. Complete Genome Sequence of Streptomyces venezuelae ATCC 15439, Producer of the Methymycin/Pikromycin Family of Macrolide Antibiotics, Using PacBio Technology. *Genome Announcements* 4. 10.1128/genomeA.00337-16

Jankowitsch F, Schwarz J, Ruckert C, Gust B, Szczepanowski R, Blom J, Pelzer S, Kalinowski J, and Mack M. 2012. Genome sequence of the bacterium Streptomyces davawensis JCM 4913 and heterologous production of the unique antibiotic roseoflavin. *Journal of Bacteriology* 194:6818-6827. 10.1128/JB.01592-12

Ohnishi Y, Ishikawa J, Hara H, Suzuki H, Ikenoya M, Ikeda H, Yamashita A, Hattori M, and Horinouchi S. 2008. Genome sequence of the streptomycin-producing microorganism Streptomyces griseus IFO 13350. *Journal of Bacteriology* 190:4050-4060. 10.1128/JB.00204-08

Omura S, Ikeda H, Ishikawa J, Hanamoto A, Takahashi C, Shinose M, Takahashi Y, Horikawa H, Nakazawa H, Osonoe T, Kikuchi H, Shiba T, Sakaki Y, and Hattori M. 2001. Genome sequence of an industrial microorganism *Streptomyces avermitilis*: deducing the ability of producing secondary metabolites. *Proceedings of the National Academy of Sciences of the United States of America* 98:12215-12220.

Ostash B, Makitrinskyy R, Walker S, and Fedorenko V. 2009. Identification and characterization of Streptomyces ghanaensis ATCC14672 integration sites for three actinophage-based plasmids. *Plasmid* 61:171-175. 10.1016/j.plasmid.2008.12.002

Ruckert C, Albersmeier A, Busche T, Jaenicke S, Winkler A, Friethjonsson OH, Hreggviethsson GO, Lambert C, Badcock D, Bernaerts K, Anne J, Economou A, and Kalinowski J. 2015. Complete genome sequence of Streptomyces lividans TK24. *Journal of Biotechnology* 199:21-22. 10.1016/j.jbiotec.2015.02.004

Thibessard A, and Leblond P. 2016. Complete Genome Sequence of Streptomyces ambofaciens DSM 40697, a Paradigm for Genome Plasticity Studies. *Genome Announcements* 4. 10.1128/genomeA.00470-16

Wang L, Gao C, Tang N, Hu S, and Wu Q. 2015. Identification of genetic variations associated with epsilon-poly-lysine biosynthesis in Streptomyces albulus ZPM by genome sequencing. *Scientific Reports* 5:9201. 10.1038/srep09201

Wang XJ, Yan YJ, Zhang B, An J, Wang JJ, Tian J, Jiang L, Chen YH, Huang SX, Yin M, Zhang J, Gao AL, Liu CX, Zhu ZX, and Xiang WS. 2010. Genome sequence of the milbemycin-producing bacterium Streptomyces bingchenggensis. *Journal of Bacteriology* 192:4526-4527. 10.1128/JB.00596-10

Zaburannyi N, Rabyk M, Ostash B, Fedorenko V, and Luzhetskyy A. 2014. Insights into naturally minimised Streptomyces albus J1074 genome. *BMC Genomics* 15:97. 10.1186/1471-2164-15-97
