# Supplementary figures and images for: The ΦBT1 large serine recombinase catalyzes DNA integration at pseudo-attB sites in the genus Nocardia
Source: PeerJ. 2018 May 4;6:e4784. doi: 10.7717/peerj.4784 (PMC5937489; doi:10.7717/peerj.4784)

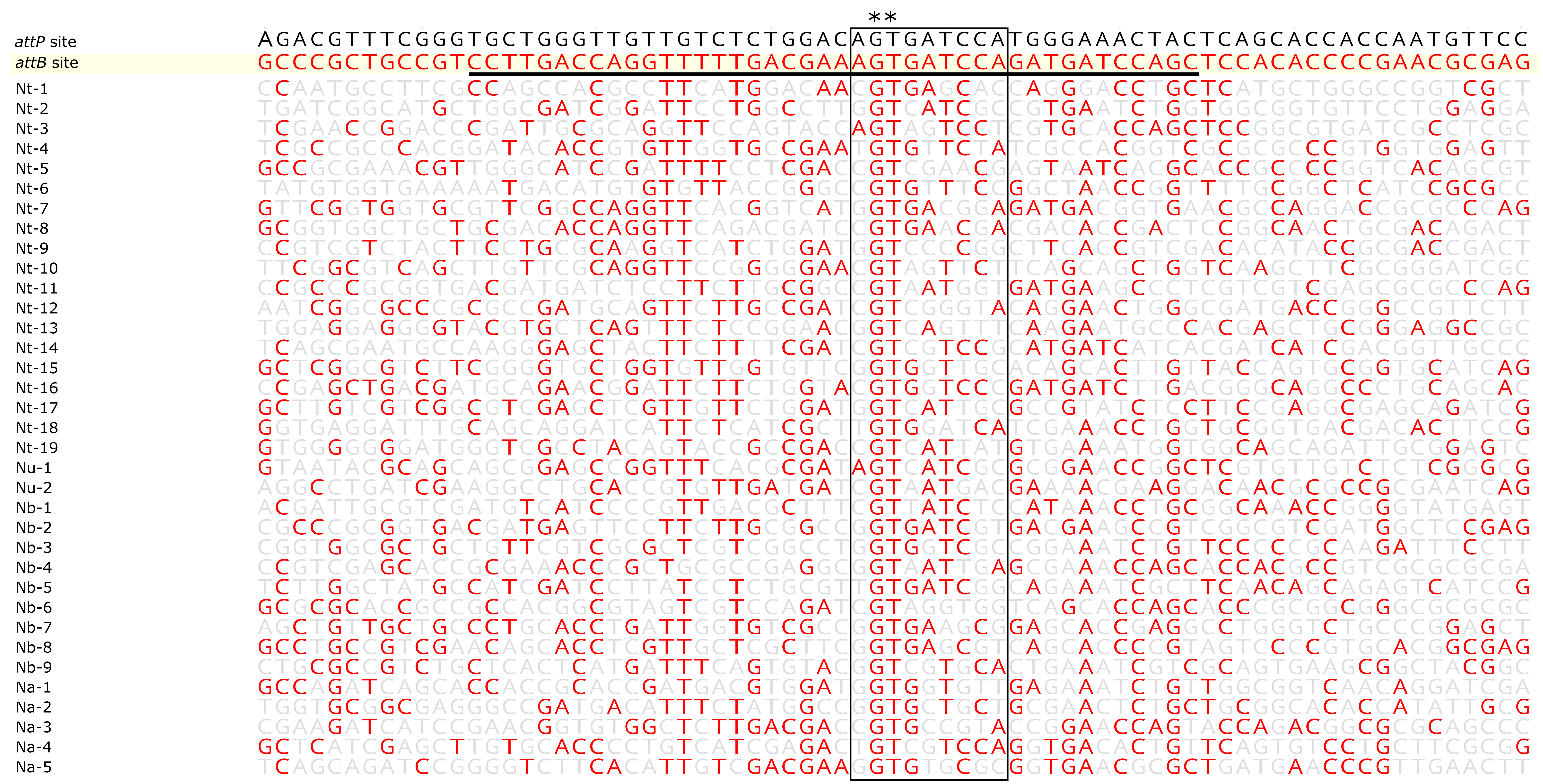

Supplement: Supplemental Information 5 — Alignment of all unique pseudo-attB insertion sites in the four tested Nocardia species compared with the S. coelicolor attB sequence. The 36 bp minimal attB is underlined and the 9 bp ΦBT1 recombination site is marked with a black box. The ΦBT1 attP site is shown in black at the top of the figure. The core GT dinucleotide is marked with asterisks. Red nucleotides represent those that are conserved with attB. [file peerj-06-4784-s005.png]

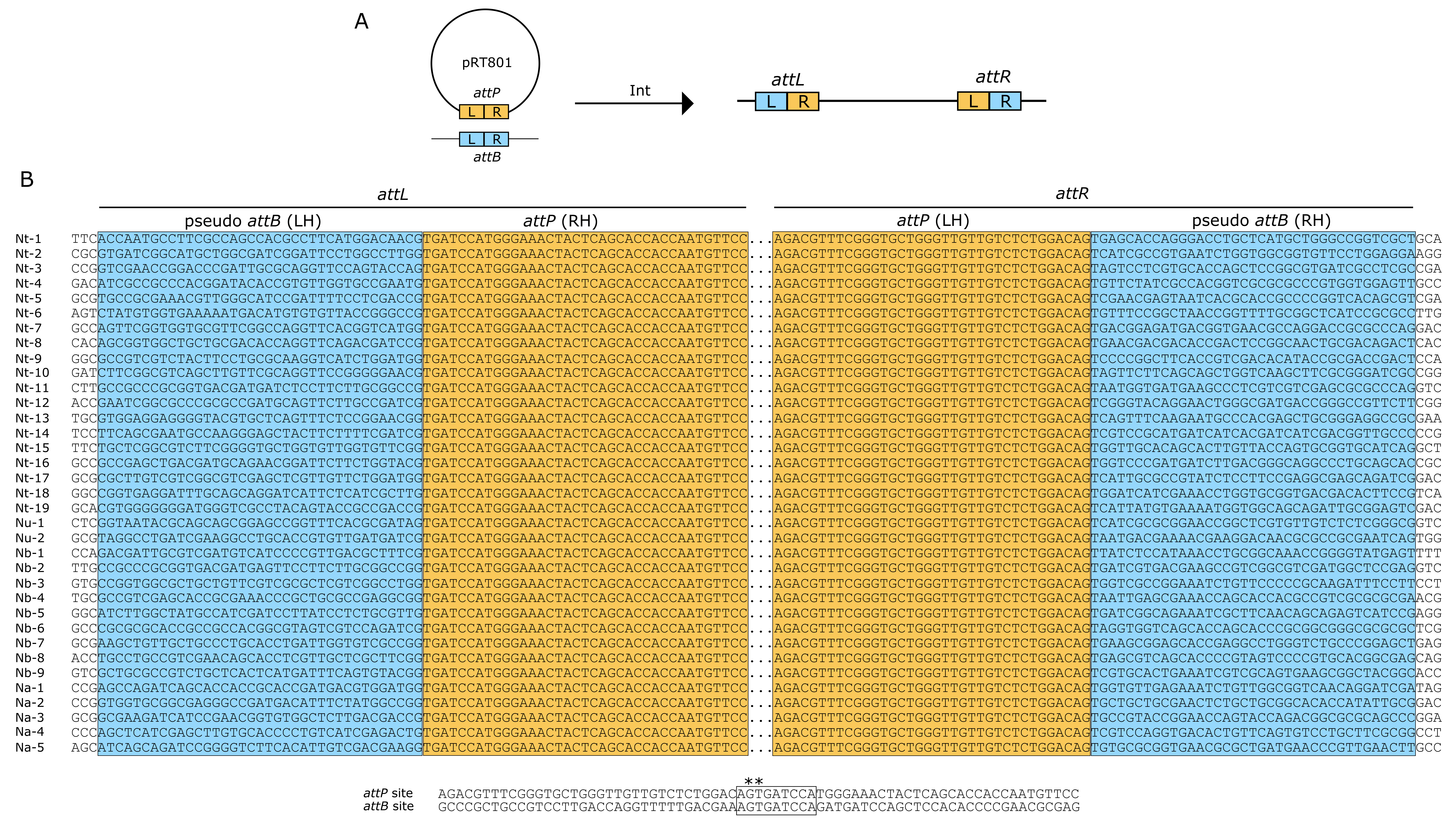

Supplement: Supplemental Information 6 — (A) Simplified diagram showing the resulting attL and attR sites that are formed following serine integrase-mediated recombination. (B) Both attL and attR sites of each unique pRT801 insertion event identified in this study are shown. Right hand and left hand arms of attB are shaded in blue, while right hand and left hand arms of attP are shaded in orange. The complete attL and attR sites are marked. The canonical attB and attP sequences for ΦBT1 from S. coelicolor are shown at the bottom, with the core GT dinucleotide indicated by asterisks and the 9bp cross-over region boxed. [file peerj-06-4784-s006.png]

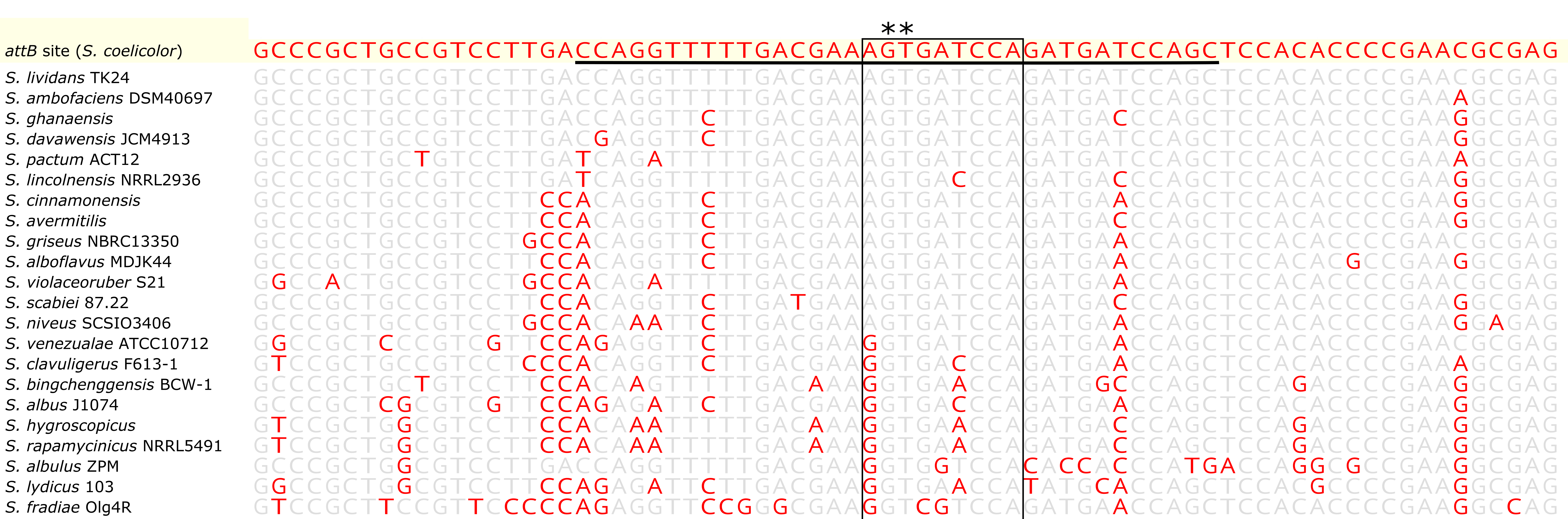

Supplement: Supplemental Information 7 — Comparison of ΦBT1 attB sites within 22 Streptomyces genomes to the canonical attB from S. coelicolor. The 36 bp minimal attB is underlined and the 9 bp ΦBT1 recombination site is marked with a black box. The core GT dinucleotide is marked with asterisks. Red nucleotides represent those that differ from the canonical attB sequence. [file peerj-06-4784-s007.png]

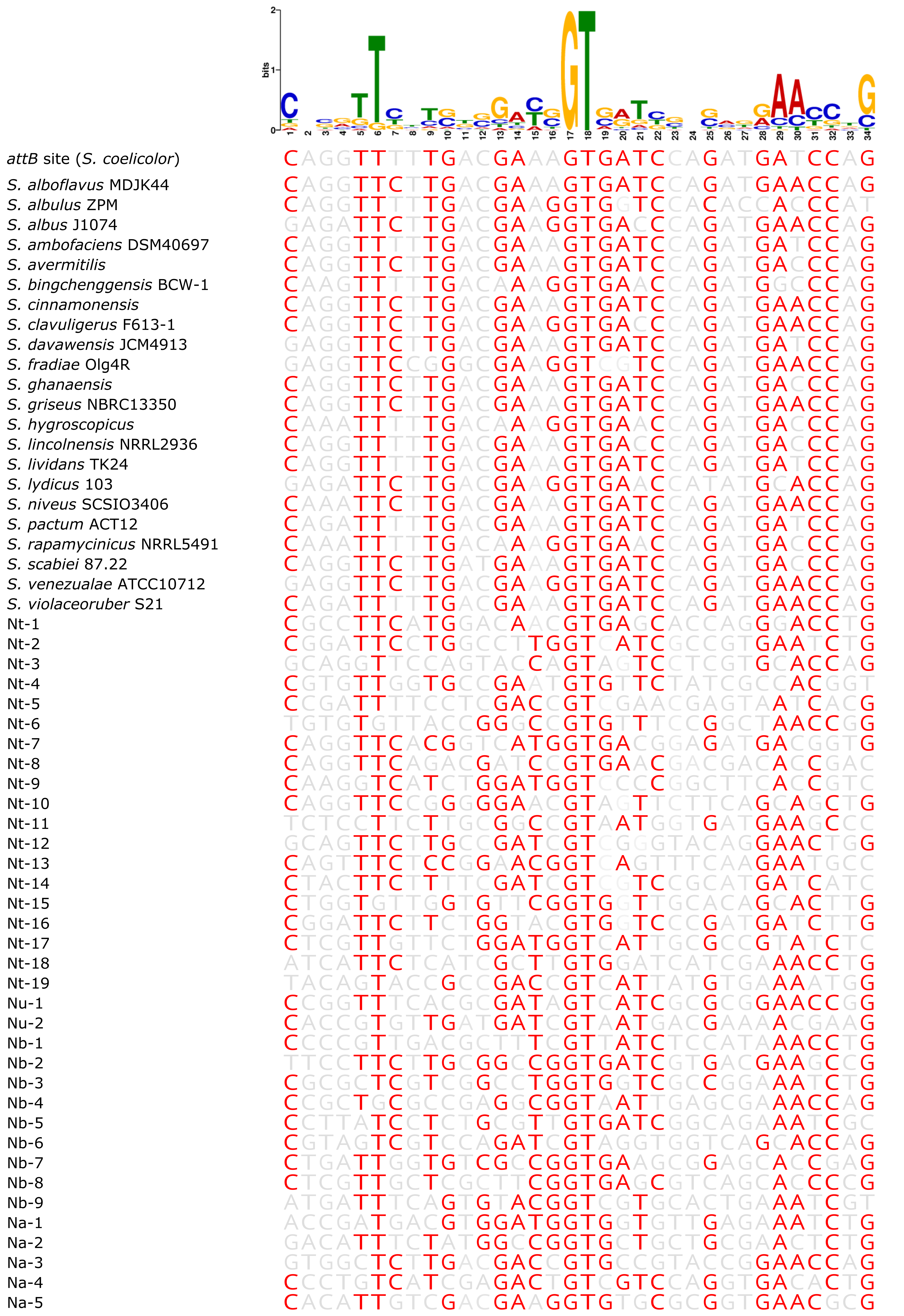

Supplement: Supplemental Information 8 — Alignment of attB sites from all analysed Nocardia and Streptomyces strains with the pseudo-attB motif. Red nucleotides are those that are identical to the most common nucleotide at each position within the predicted motif. [file peerj-06-4784-s008.png]
